# Effect of renal function on high-density lipoprotein particles in patients with coronary heart disease

**DOI:** 10.1186/s12872-021-02354-2

**Published:** 2021-11-12

**Authors:** Long Jieni, Xue Yazhi, Zeng Xiaorong, Liu Dan, Ma Yusheng, Rao Jiahuan, Zhang Bin, Li Li, Guo Zhigang

**Affiliations:** 1 Huiqiao Medical Center, Standardized General Practice Training Site, Nanfang Hospital, Southern Medical University, Guangzhou, 510515 Guangdong Province China; 2 Department of Cardiology, Nanfang Hospital, Southern Medical University, Guangzhou, 510515 Guangdong Province China

**Keywords:** Coronary heart disease, Renal insufficiency, HDL-C/apoA-I, HDL particles

## Abstract

**Background:**

Although renal insufficiency and dyslipidemia are known to be closely associated, the effect of kidney function on the size and clinical value of high-density lipoprotein (HDL) particles remains largely unknown, especially in patients with coronary heart disease.

**Methods:**

A total of 419 coronary heart disease patients and 105 non-coronary heart disease patients were included. HDL particle size, represented by HDL-C/apoA-I, was compared between groups stratified by estimated glomerular filtration rate (eGFR) and Gensini scores using standard Student’s t test and one-way ANOVA. Pearson’s correlation test was performed to analyze the association between eGFR and HDL-C/apoA-I in patients with coronary heart disease. The relationship between HDL particle size and the occurrence of coronary heart disease was explored using Univariate logistic regression analysis.

**Results:**

In patients with coronary heart disease, between-group analysis revealed that HDL-C/apoA-I increased as eGFR declined, and significance appeared as eGFR declined to under 60 ml/min·1.73 m^2^ (*P* < 0.001), and Pearson's correlation test also confirmed an inverse correlation between eGFR and HDL-C/apoA-I levels in coronary heart disease patients. When stratified by Gensini scores, in coronary heart disease patients with eGFR ≥ 90 mL/(min·1.73 m^2^), those with higher Gensini scores had smaller HDL-C/apoA-I. However, with or without kidney insufficiency, smaller HDL-C/apoA-I was associated with a higher occurrence of coronary heart disease (*P* < 0.05).

**Conclusion:**

With the presence of renal insufficiency, HDL-C/apoA1 was higher in patients with coronary heart disease. Lower HDL-C/apoA1 was still associated with a higher occurrence of coronary heart disease, but the original association between lower HDL-C/apoA1 and more severe coronary artery stenosis was lost in patients with renal insufficiency.

## Background

High-density lipoprotein (HDL) has been acknowledged as a protective factor for cardiovascular disease, as there is unequivocal evidence of the inverse association between the HDL-C level and the occurrence and mortality of cardiovascular disease [1]. However, in recent years, several clinical trials aiming to increase plasma high-density lipoprotein cholesterol (HDL-C) concentrations, such as the ILLUMINATE study, AIM-HIGH study and HPS2-THRIVE study, have failed to consistently obtain satisfying results, indicating that HDL particles and HDL function may play a more important role than the HDL-C level in plasma [2–4]. HDL lipoproteins are in fact not intact particles, but consisting of components that differ in composition and function, yet the exact relationship between HDL particles, HDL function and cardiovascular diseases remains unclear.

Dyslipidemia is also closely associated with renal insufficiency. Chronic kidney disease is believed to be related to systemic inflammatory responses, and in recent years, evidence has shown that dyslipidemia may contribute to the development of kidney dysfunction [5]. Alterations in the distribution and quality of lipoproteins have been reported in multiple studies, but the findings appear to be inconsistent and even contradictory due to the complex nature of lipid metabolism. Large, mature HDL particles are generally considered to be anti-inflammatory and cardiovascular protective, and recently, studies have reported decreased amounts of larger HDL particles in patients with end stage kidney disease (ESRD) [6].

Due to the complicated compositions of HDL, nuclear magnetic resonance imaging (NMRI) is usually used to measure the diameter of HDL particles [7]; however, this specific method can be difficult to apply. In 2013, in a study based on the Women's Health study, the ratio of HDL-C to apolipoprotein A-I (apoA-I) was discovered to be linearly correlated with actual HDL diameter measured by NMRI, indicating that HDL-C/apoA-I can serve as a reliable biomarker representing HDL particle size [8].

In this study, the potential effect of kidney function on the value and size of HDL, represented by HDL-C/apoA-I, was explored in patients with coronary heart disease.

## Methods

### Study populations

A total of 524 inpatients met the following inclusion criteria were enrolled by simple random sampling: (1) Hospitalized and underwent underwent coronary angiography for the first time between January 2013 and December 2018; (2) No lipid-lowering medications in 3 months prior to hospitalization; (3) All necessary data were available. Exclusion criteria: (1) Previous history of percutaneous coronary intervention or coronary artery bypass grafting; (2) Acute kidney injury or on dialysis; (3) Other diseases that might cause unstable hemodynamics such as severe infectious diseases, hematologic diseases and progressive tumors. The Study was approved by and carried out in accordance with institutional review board of Nanfang Hosiptal of Southern Medical University, and informed consent was obtained from participants/legal guardians of participants involved in the study.

### Laboratory measurement and calculation

Fasting venues blood samples were collected at the time of admission and stored at − 70 °C after centrifugation and tested with standard measurements in the laboratory of Nanfang Hospital. CR (Creatinine), Cys-C (Cystatin C), CRP (C-reactive protein), TG (Triglyceride), TC (Total Cholesterol), HDL-C, LDL-C (Low density lipoprotein cholesterol), apoA-I, apoB (Apolipoprotein B), apoE (Apolipoprotein E) and Lp(a) (Lipoprotein (a)) were all measured by Beckman Coulter AU5431.

### Statistical analysis

Patients with an estimated glomerular filtration rate(eGFR) lower than 90 ml/min·1.73 m^2^ were defined as having renal insufficiency. All data were collected directly from medical records, and blood samples were collected within 24 h after admission. The Gensini scoring system was used to evaluate coronary angiography, interpreted by 2 experienced coronary angiography operators. The Chronic Kidney Disease Epidemiology Collaboration (CKD-EPI) formula was used to estimate the glomerular filtration rate. HDL-C/apoA-I was used to represent HDL particle size.

SPSS 20.0 was used for statistical analysis. Descriptive findings are presented as the mean and standard deviation (Mean ± SD), proportions (%) or interquartile range (IQR 25–75%). Data comparison between two groups was performed by the standard Student’s t test; comparisons between multiple groups wereperformed by one-way ANOVA. For counting data, the chi-square test was performed. Pearson’s correlation test was performed to analyze the association between eGFR and HDL-C/apoA-I in patients with coronary heart disease. Univariate logistic regression analysis was used to explore the association between HDL-C/apoA-I and risk for the occurrence of coronary heart disease. All p values were 2-tailed.

## Results

### Baseline characteristics and lipid profiles between patients with and without coronary heart disease

A total of 419 coronary heart disease patients and 105 non-coronary heart disease patients were included in our study. As shown in Table 1, patients with coronary heart disease tended to be smokers (50.12% vs. 36.19%, *P* = 0.008). HDL-C and apoA-I levels both decreased significantly in patients with coronary heart disease (0.96 ± 0.01 vs. 1.09 ± 0.03, *P* < 0.001 and 1.12 ± 0.01 vs. 1.18 ± 0.02, *P* < 0.001, respectively), along with HDL-C/apoA-I (0.85 ± 0.13 vs. 0.92 ± 0.20, *P* = 0.005), while Lp(a) levels were shown to increase in coronary heart disease patients (0.31 ± 0.01 vs. 0.25 ± 0.02, *P* = 0.001).

**Table 1:** Baseline characteristics and lipid profiles in patients with and without coronary heart disease

	Coronary heart disease (n = 419)	Noncoronary heart disease (n = 105)	*P* value
eGFR (ml/min·1.73 m^2^)	84.88 ± 24.61	79.88 ± 30.356	0.105
Sex (male/female)	328/91	77/28	0.170
Age (years)	58.72 ± 11.02	57.16 ± 12.67	0.26
BMI (kg/m^2^)	24.34 ± 0.24	24.48 ± 0.47	0.777
Smoking (%)	50.12	36.19	0.008
Hypertension (%)	56.56	62.86	0.270
Diabetes (%)	24.10	20.00	0.439
Systolic BP when Admitted (mmHg)	134.18 ± 37.24	133.05 ± 24.68	0.768
Diastolic BP when Admitted (mmHg)	79.89 ± 13.72	80.74 ± 14.97	0.574
CR (*ч*mol/L)	90.47 ± 31.53	95.64 ± 40.00	0.157
Cys-C(mmol/L)	1.27 ± 0.07	1.10 ± 0.03	0.513
CRP (mg/L)	16.51 ± 1.65	15.07 ± 2.89	0.681
TG (mmol/L)	1.79 ± 0.05	1.63 ± 0.10	0.195
TC (mmol/L)	4.62 ± 0.06	4.64 ± 0.12	0.838
HDL-C (mmol/L)	0.96 ± 0.01	1.09 ± 0.03	0.000
LDL-C (mmol/L)	2.93 ± 0.04	2.92 ± 0.09	0.905
apoA-I (g/L)	1.12 ± 0.01	1.18 ± 0.02	0.001
apoB (g/L)	1.06 ± 0.10	0.96 ± 0.02	0.615
Lp(a) (g/L)	0.31 ± 0.01	0.25 ± 0.02	0.012
apoE (mg/L)	46.64 ± 0.80	46.27 ± 1.48	0.835
HDL-C/apoA-I	0.85 ± 0.13	0.92 ± 0.20	0.005

BMI, Body mass index; BP, Blood Pressure

### Lipid profiles and gensini scores in patients with coronary heart disease, stratified by eGFR

**Fig. 1:**
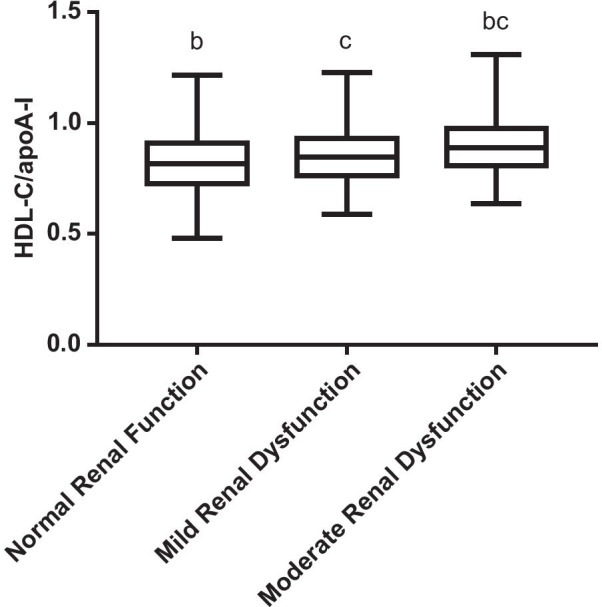
HDL-C/apoA-I stratified by chronic kidney disease categories in patients with coronary heart disease. a Indicatesa significant difference between patients with normal kidney function and patients with mildly reduced kidney function (60 ml/min·1.73 m^2^≦eGFR < 90 ml/min·1.73 m^2^); b indicatesa significant difference between patients with normal kidney function and patients with moderately reduced kidney function (eGFR≦60 ml/min·1.73 m^2^); c indicates a significant difference between patients with mildly reduced kidney function and patients with moderately reduced kidney function

**Fig. 2:**
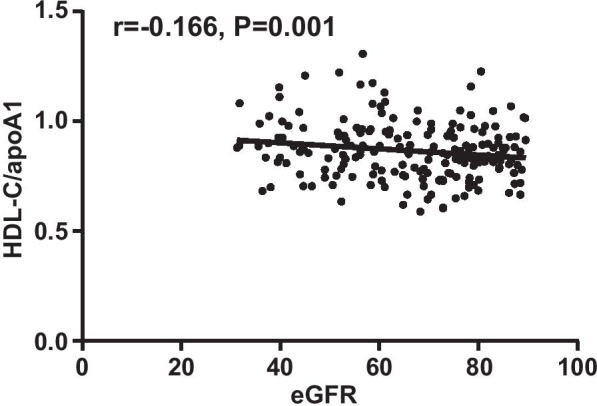
Correlation of eGFR and HDL-C/apoA-I in coronary heart disease patients

As shown in Table 2, in patients with coronary heart disease, 227 patients had decreased eGFR (eGFR < 90 ml/min·1.73 m^2^). Both HDL-C/apoA-I levels and HDL-C levels were notably higher in patients with kidney insufficiency (0.83 ± 0.13 and 0.87 ± 0.11, *P* = 0.005 and0.93 ± 0.02 and 0.98 ± 0.02, *P* = 0.024).Additionally, patients were grouped by chronic kidney disease categories, and between-group analysis revealed that HDL-C/apoA-I increased as eGFR declined, and significance was presented in patients with moderately reduced kidney function (eGFR < 60 ml/min·1.73 m^2^; *P* < 0.001), as shown in Table 3 and Fig. 1. Finally, Pearson's correlation test also confirmed an inverse correlation between eGFR and HDL-C/apoA-I levels in coronary heart disease patients, as shown in Fig. 2 (r = -0.166, *P* = 0.001).

**Table 2:** Baseline characteristics, lipid profiles and gensini scores in coronary heart disease patients with normal kidney function and kidney insufficiency

	Normal Kidney Function (n = 192)	Renal insufficiency (n = 227)	*P* value
eGFR (ml/min·1.73 m^2^)	106.30 ± 17.00	67.81 ± 14.14	0.000
Sex(Male/Female)	138/54	190/37	0.003
Age	57.72 ± 9.71	59.60 ± 12.00	0.082
BMI (kg/m^2^)	23.91 ± 0.33	24.56 ± 0.32	0.165
Smoking (%)	53.14	47.14	0.240
Hypertension (%)	45.31	58.15	0.490
Diabetes (%)	28.65	20.26	0.052
Systolic BP when Admitted (mmHg)	133.54 ± 21.77	134.72 ± 46.56	0.747
Diastolic BP when Admitted (mmHg)	79.83 ± 13.40	79.93 ± 14.00	0.943
CR (*ч*mol/L)	71.93 ± 11.41	106.15 ± 34.49	0.000
Cys-C (mmol/L)	1.06 ± 0.03	1.43 ± 0.13	0.014
CRP (mg/L)	16.26 ± 2.59	16.72 ± 2.11	0.890
TG (mmol/L)	1.82 ± 0.08	1.76 ± 0.07	0.594
TC (mmol/L)	4.51 ± 0.09	4.71 ± 0.08	0.074
HDL-C (mmol/L)	0.93 ± 0.02	0.98 ± 0.02	0.024
LDL-C (mmol/L)	2.86 ± 0.07	2.99 ± 0.06	0.122
apoA-I (g/L)	1.11 ± 0.01	1.13 ± 0.01	0.415
apoB (g/L)	1.15 ± 0.22	0.98 ± 0.02	0.386
Lp(a) (g/L)	0.29 ± 0.02	0.33 ± 0.02	0.157
apoE (mg/L)	47.01 ± 1.24	46.32 ± 1.04	0.665
HDL-C/apoA-I	0.83 ± 0.13	0.87 ± 0.11	0.005
Gensini Scores	56.40 ± 3.11	59.11 ± 3.09	0.539

**Table 3:** Baseline characteristics, lipid profiles and gensini scores in coronary heart disease patients stratified by chronic kidney disease categories

	Normal Kidney Function (n = 192)	eGFR (ml/min·1.73 m^2^)		*P* value
		60–90 (n = 160)	≤ 60 (n = 67)	
eGFR (ml/min·1.73 m^2^)	106.30 ± 17.00^ab^	74.74 ± 8.88^ac^	50.26 ± 8.76^bc^	0.000
Sex(Male/Female)	138/54	135/25	55/12	0.013
Age	57.72 ± 9.70^b^	58.66 ± 12.36^bc^	62.17 ± 10.64^bc^	0.026
CR (*ч*mol/L)	71.93 ± 11.42^ab^	93.30 ± 14.56^ac^	136.82 ± 46.97^bc^	0.000
TG (mmol/L)	1.82 ± 0.08	1.78 ± 0.08	1.73 ± 0.13	0.832
TC (mmol/L)	4.51 ± 0.09	4.74 ± 0.10	4.65 ± 0.12	0.175
HDL-C (mmol/L)	0.93 ± 0.02^a^	0.96 ± 0.02^a^	1.03 ± 0.03	0.014
LDL-C (mmol/L)	2.86 ± 0.07	3.03 ± 0.07	2.91 ± 0.10	0.203
apoA-I (g/L)	1.11 ± 0.01^ab^	1.13 ± 0.01^ac^	1.11 ± 0.02^bc^	0.713
apoB (g/L)	1.15 ± 0.22	0.99 ± 0.02	0.95 ± 0.03	0.679
Lp(a) (g/L)	0.29 ± 0.02^ab^	0.33 ± 0.03^ac^	0.33 ± 0.04^bc^	0.367
apoE (mg/L)	47.01 ± 1.24	46.33 ± 1.24	46.28 ± 1.93	0.910
HDL-C/apoA-I	0.83 ± 0.13^b^	0.85 ± 0.11^c^	0.90 ± 0.13^bc^	0.000
Gensini Scores	56.40 ± 3.11^b^	57.74 ± 3.49^c^	62.40 ± 6.34^bc^	0.644

Aindicatesa significant difference between patients with normal kidney function and patients with mildly reduced kidney function (60 ml/min·1.73 m^2^≦eGFR < 90 ml/min·1.73 m^2^); b indicatesa significant difference between patients with normal kidney function and patients with moderately reduced kidney function (eGFR≦60 ml/min·1.73 m^2^); c indicates a significantdifference between patients with mildly reduced kidney function and patients with moderately reduced kidney function

### HDL-C/apoA-I in patients with coronary heart disease stratified by gensini scores

Coronary heart disease patients with decreased eGFR were grouped by the median Gensini score, and HDL-C/apoA-I was compared between them (Table 4). In patients with more severe coronary artery stenosis (n = 112), HDL-C/apoA-I levels were presented to be decreasing, but no statistical significance was observed (P = 0.875) between patients with different severities of stenosis. Thus, the same method was applied in all enrolled subjects and coronary heart disease patients without kidney insufficiency, and in both groups, HDL-C/apoA-I levels increased significantly higher with lower Gensini scores (0.849 ± 0.127 and 0.877 ± 0.177, respectively, in all enrolled patients, *P* = 0.044; 0.816 ± 0.133 and 0.849 ± 0.129, respectively, in coronary heart disease patients without kidney insufficiency, *P* = 0.007).

**Table 4:** HDL-C/apoA-I in patients with coronary heart disease stratified by gensini scores

	HDL-C/apoA-I	*P* value
All enrolled patients		
High Gensini scores	0.849 ± 0.127	0.044
Low Gensini scores	0.877 ± 0.177	
Coronary heart disease patient with kidney insufficiency		
High Gensini scores	0.866 ± 0.113	0.875
Low Gensini scores	0.869 ± 0.112	
Coronary heart disease patient without kidney insufficiency		
High Gensini Scores	0.816 ± 0.133	0.007
Low Gensini Scores	0.849 ± 0.129	

### Association of HDL-C/apoA-I and the occurrence of coronary heart disease

To analyze the association of HDL-C/apoA-I with the occurrence of coronary heart disease in different groups, HDL-C/apoA-I was stratified by median with the higher group as a reference, and then logistic regression analysis was applied in patients with normal kidney function and patients with declined eGFR (Table 5). Regardless of kidney function, higher HDL-C/apoA-I was associated with a lower risk for coronary heart disease (OR 0.378 and OR 0.349, *P* < 0.05).

**Table 5:** Association of HDL-C/apoA-I with the occurrence of coronary heart disease

	OR	95% CI	*P* value
Patients without kidney insufficiency			
Low HDL-C/apoA-I	1		
High HDL-C/apoA-I	0.478	0.230–0.994	0.048
Patients with kidney insufficiency			
Low HDL-C/apoA-I	1		
High HDL-C/apoA-I	0.349	0.423–1.258	0.000

## Discussion

Dyslipidemia is an independent risk factor for cardiovascular disease, which is the leading cause of death in chronic kidney disease patients [9]. Renal deficiency has also been identified as an independent risk factor for coronary heart disease, as kidney deficiency may contribute to the development of atherosclerosis and is related to premature cardiovascular events [10]. Stack [11] et al. have reported that patients on hemodialysis had a 38% higher incidence of cardiovascular diseases, and other studies have also reported that for every 10 mL/(min·1.73 m^2^) reduction in eGFR, the occurrence of cardiovascular diseases can increase by 19% [12].

HDL has been widely acknowledged as a protective factor in both cardiovascular disease and chronic kidney disease patients, as it acts as an acceptor of cellular cholesterol and initiates reverse cholesterol transport by transferring peripheral cholesterol back to the liver. Lower plasma HDL-C levels are known to be associated with an increased occurrence of major cardiovascular events.The Framingham study has reported that in a healthy population, for every 1 mg/dl increase in HDL-C levels, the incidence of coronary heart disease was reduced by 2% to 3% [1]. However, in recent years, studies aimed at elevating HDL-C levels have successively failed to reduce cardiovascular events, and it has been reported that high HDL-C levels are not always correlated with reduced cardiovascular death [13]. Therefore, an increasing number of studies have shifted their focus from the HDL-C level to HDL function and HDL particle size.

HDL is mainly composed of larger HDL2 and smaller HDL3. In the process of reverse cholesterol transport (RCT), small, flat pre-β-HDL gradually matures into large, spherical HDL2; thus, larger HDL particles are generally considered to be more mature and have greater cardiovascular protective effects [14]. In cardiovascular disease, the subpopulations of HDL may be altered due to a disrupted or even hindered RCT process [15]. Both HDL dysfunction and altered HDL distribution are likely to be related to oxidative stress and inflammation [16, 17], which also contribute to endothelial dysfunction and account for their association with the acceleration of chronic kidney disease.

In our study, lower HDL-C/apoA1 was shown to be closely related to coronary artery stenosis in coronary heart disease patients with normal kidney function; however, in coronary heart disease patients with a moderately decreased eGFR, HDL-C/apoA-1 grew larger, and the original association with coronary artery stenosis was also lost. Since HDL-C/apoA-I was used to represent HDL particle sizes in our study, our results demonstrated the same association between HDL particle sizes and coronary artery stenosis. Similar phenomenons has been reported in several studies [18–20]. In the past, it was believed that lipoproteins cannot pass the glomerular filtration barrier. Despite HDL being the smallest of all lipoproteins, containing the least lipids but the most protein, there is no evidence of intact HDL particles filtering through the glomerular filtration barrier. However, subfractions of HDL particles metabolize separately^18^. Albumin, which has a molecular weight of 66.5 kD, has already been proven to cross the glomerular filtration barrier,; thus, apoA-I, weighted 28 kD, and pre-β-HDL, weighted 60 to 85 kD, can both cross a normally functioning glomerular filtration barrier theoretically, while larger subfractions are likely to be filtered in individuals with injured kidney function [21]. Therefore, it is reasonable to assume that increased HDL particle size in chronic kidney disease patients is a result of the loss of smaller HDL particles through the glomerular filtration barrier.

Our results also reported the loss of association between HDL particle size and Gensini score in the context of comorbidity of coronary heart disease with renal insufficiency. In our study, renal defficiency was defined as an eGFR < 90 mL/(min·1.73 m^2^), as renal damage and related dyslipidemia have already started in early stages of chronic kidney disease [22]. Chronic kidney disease is known to be an independent risk factor for cardiovascular disease, so it is reasonable to assume that with renal insufficiency in the picture, the size shifting of HDL particles, which reflects only an imbalance in HDL metabolism, is no longer a strong predictor of the severity of coronary heart disease [23, 24]. In coronary heart patients with renal insufficiency, the protective effects of larger HDL particles may be weakened; thus, lipid-targeting therapy may not be as essential in these patients.

Our study explored the still-debated effect of renal function on HDL particle size in patients with coronary artery disease using HDL-C/apoA-I to represent HDL particle size. However, as a single-center retrospective study, the sample size was relatively small, and further confirmation of our theory would require analysis of urine protein profiles. Additionally, HDL-C/apoA-I represents only the average size of HDL particles; thus, HDL subfraction analysis is still needed if the specific alterations in HDL subpopulations in chronic kidney disease patients are to be explored in the future.

## Conclusion

In our study, lower HDL-C/apoA-1was shown to be associated with more severe coronary artery stenosis in coronary heart disease patients with normal kidney function. However, in coronary heart disease patients with renal insufficiency, an inverse correlation of eGFR with HDL-C/apoA-1was found as eGFR declined to 60 ml/min·1.73 m^2^, accompanied by a loss of association between HDL-C/apoA-I and coronary artery stenosis.

## Data Availability

The data that support the findings of this study are available from Southern Medical University Nanfang Hospital but restrictions apply to the availability of these data, which were used under license for the current study, and so are not publicly available. Data are however available from the authors upon reasonable request and with permission of Southern Medical University Nanfang Hospital.

## Abbreviations

HDL: High-density lipoprotein
HDL-C: High-density lipoprotein cholesterol
apoA-I: Apolipoprotein A-I
eGFR: Estimated glomerular filtration rate
ESRD: End-staged kidney disease
NMRI: Nuclear magnetic resonance imaging
CKD-EPI: The Chronic Kidney Disease Epidemiology Collaboration
BMI: Body mass index
BP: Blood Pressure
CR: Creatinine
Cys-C: Cystatin C
CRP: C-reactive protein
TG: Triglyceride
TC: Total Cholesterol
apoB: Apolipoprotein B
Lp(a): Lipoprotein (a)
apoE: Apolipoprotein E
